# Non-specific low back pain and its relation to the nursing work process *

**DOI:** 10.1590/1518-8345.2915.3172

**Published:** 2019-10-07

**Authors:** Zulamar Aguiar Cargnin, Dulcinéia Ghizoni Schneider, Mara Ambrosina de Oliveira Vargas, Rosani Ramos Machado

**Affiliations:** 1 Universidade Federal de Santa Catarina , Florianopólis , SC , Brasil .; 2 Universidade Federal de Santa Catarina , Departamento de Enfermagem , Florianópolis , SC , Brasil .

**Keywords:** Low Back Pain, Nursing Process, Working Conditions, Occupational Health, Workload, Nursing, Dor Lombar, Processo de Enfermagem, Condições de Trabalho, Saúde do Trabalhador, Carga de Trabalho, Enfermagem, Dolor de la Región Lumbar, Proceso de Enfermería, Condiciones de Trabajo, Salud Laboral, Carga de Trabajo, Enfermería

## Abstract

**Objective:**

to relate nonspecific low back pain within the nursing work context with their workloads, attrition processes and the risks of illness.

**Method:**

a cross-sectional study with 301 workers from a general hospital in the south of the country. The Nordic Musculoskeletal Questionnaire and the Work Context Assessment Scale composed of three dimensions were used: working conditions, work organization and socio-professional relations. The association of variables with low back pain was tested using bivariate and multivariate analyzes. The measure of association used was the Odds Ratio and its respective intervals with 95% confidence. The data collected were discussed under the theoretical framework of the work process within the marxist conception and the theory of social determination of the health-disease process.

**Results:**

there was a statistically significant association between the dimensions of work organization and working conditions with low back pain and they obtained a critical classification meaning moderate risks to the professional illness.

**Conclusion:**

the study allowed a better understanding of the nursing work process and its relation with nonspecific low back pain and signaled that changes in the organization and working conditions should occur in order to reduce the risks of nursing workers’ illness.

## Abstract (pt)

**Objetivo:**

relacionar a dor lombar inespecífica dentro do contexto de trabalho da enfermagem com suas cargas de trabalho, processos de desgaste e os riscos de adoecimento.

**Método:**

estudo transversal com 301 trabalhadores de um hospital geral do Sul do país. Utilizaram-se o *Nordic Musculoskeletal Questionnaire* e a Escala de Avaliação do Contexto de Trabalho composta de três dimensões: “condições de trabalho”, “organização do trabalho” e “relações socioprofissionais”. Foi testada a associação das variáveis com a dor lombar por meio de análises bivariadas e multivariadas. A medida de associação utilizada foi a *Odds Ratio* e seus respectivos intervalos com 95% de confiança. Os dados coletados foram discutidos sob o referencial teórico do processo de trabalho dentro da concepção marxista e da Teoria da Determinação Social do Processo Saúde-doença.

**Resultados:**

houve associação estatisticamente significativa entre as dimensões “organização do trabalho” e “condições de trabalho com a dor lombar”, que obtiveram classificação crítica, significando riscos moderados ao adoecimento profissional.

**Conclusão:**

o estudo permitiu maior compreensão do processo de trabalho de enfermagem e sua relação com a lombalgia inespecífica e sinalizou que devem ocorrer mudanças na organização e nas condições de trabalho a fim de diminuir os riscos de adoecimento dos trabalhadores de enfermagem.

## Abstract (es)

**Objetivo:**

relacionar el dolor lumbar inespecífico dentro del contexto de trabajo de la enfermería con sus cargas de trabajo, procesos de desgaste y los riesgos de enfermedad.

**Método:**

estudio transversal con 301 trabajadores de un hospital general del Sur del país. Se utilizaron el *Nordic Musculoskeletal Questionnaire* y la Escala de Evaluación del Contexto de Trabajo compuesta de tres dimensiones: “condiciones de trabajo”, “organización del trabajo” y “relaciones socioprofesionales”. Se probó la asociación de las variables con el dolor lumbar por medio de análisis bivariados y multivariados. La medida de asociación utilizada fue la *Odds Ratio* y sus respectivos intervalos con un 95% de confianza. Los datos recolectados fueron discutidos bajo el referencial teórico del proceso de trabajo dentro de la concepción marxista y de la Teoría de la Determinación Social del Proceso Salud-enfermedad.

**Resultados:**

hubo asociación estadísticamente significativa entre las dimensiones “organización del trabajo” y “condiciones de trabajo con el dolor lumbar”, que obtuvieron clasificación crítica, significando riesgos moderados a la enfermedad profesional.

**Conclusión:**

el estudio permitió una mayor comprensión del proceso de trabajo de enfermería y su relación con la lumbalgia inespecífica y señaló que deben ocurrir cambios en la organización y en las condiciones de trabajo a fin de disminuir los riesgos de enfermarse de los trabajadores de enfermería.

## Introduction

Work is an exchange between man and nature that transforms and expresses a purpose. At the same time, exploitation of the labor force can occur through its organization and division, whose economic factors exert a positive or negative influence ^1 - 2^ . It is necessary to understand the social character of the health-disease binomial in connection with the production system/medium. The labor process is both social and biopsychic. Identifying the cause of the injuries in this scenario is a social practice in which, in the community, the factors and determinants responsible for the profile of workers’ illnesses are sought out ^3 - 4^ .

The professionals, who work in the area of nursing, are exposed to different loads that compromise their life and health ^4^ . These charges interact with each other and with the worker’s body, which establishes responses to the global labor process ^5 - 6^ . Therefore, occupational diseases, wear and tear, absenteeism and accidents that generate financial costs and decrease in productivity, quality and safety of care ^4 , 7^ . Nursing is the focus of research, mainly at the hospital level, due to the adverse conditions of its activities and the exposure to various loads that interrelate with each other and with the organization of tasks ^8 - 9^ .

Low back pain is located in the lumbar region between the last costal arch and the gluteal fold ^10^ . In turn, it is non-specific, when it does not have a specific and well-determined diagnosis, besides corresponding to about 90 to 95% of the cases ^11 - 12^ . It is part of an occupational disease that constitutes a worldwide public health problem due to its high prevalence. It affects all age groups and socioeconomic levels and requires promotion, education and prevention, not just health rehabilitation with effective global initiatives ^11 - 12^ .

Factors related to low back pain are multiple and complex ^11 - 13^ . Professional risks involve the vision of the work context, the physical demands, the ergonomic, psychosocial factors and the forms of organization and execution of the tasks ^13^ .

This study aimed to relate nonspecific low back pain within a nursing work context with its workloads, attrition processes and the risks of illness. For this, the interrelationship of aspects of the labor process involving its conditions, organization and socio-professional relations were analyzed.

## Method

Cross-sectional study carried out in a public hospital of medium and high complexity in Florianópolis, linked to the State Health Department, with diverse specialties. At the time of data collection, it had 225 active beds. The place was chosen due to the multiplicity of specialties, besides having an expressive contingent of nursing professionals.

It was discussed about the theoretical reference of the Marxist conception ^1^ and the theory of social determination of the health-disease process based on workloads from a 1989 study ^3 - 4^ . Both were based on historical and dialectical materialism that reflects reality and social dynamics to explain the health-disease process, interconnecting, in this case, non-specific lower back pain. It is considered that the work process and the productive forces influence the pathological profile of nursing, since this profession is exposed to different workloads. These, for this study, are defined as the interrelationship of factors with the worker’s body that cause wear and tear and concern the conditions, organization and division of labor that permeate the care work ^4 , 14^ . The work loads can be grouped in physical, chemical, biological and mechanical that have external materiality, observed directly. As well, they can be divided into physiological and psychic that are of internal materiality because they only acquire materiality in the human body by means of transformations in their internal processes and they are manifested by means of disorder or disease ^4 , 6 - 7 , 14^ .

All the auxiliaries, nursing technicians and nurses who were working in the Hospital during the period of data collection, characterizing a census of 353 workers, were invited to participate in the study. After applying the inclusion and exclusion criteria, the final sample consisted of 301 nursing workers, characterizing 87.8% of the population invited to participate in the study.

Professionals who work exclusively in nursing and who carry out their activities for at least one year were included. This time was selected to establish a possible link with work activity as a contributor to low back pain. Patients with low back pain with specific diagnoses such as spondylolisthesis, disc herniation, spinal canal stenosis, infectious spinal diseases, spine tumors and fractures were excluded.

Data collection was carried out from May to October 2017. The workers were individually approached during their work shift, in all sectors of the hospital, using the service scales, including the morning, afternoon and evening shifts. Each questionnaire self-administered questionnaire explained the objectives of the research, its importance, the confidentiality of the information, the anonymity of the participants, completion instructions and, through the participation of the research, a date was set for the devolution and validation of the questionnaires by the participants.

The data collection was done through a multidimensional form with open and closed questions, related to sociodemographic and labor data, constructed by the authors; the Nordic Musculoskeletal Questionnaire (NMQ) and the Work Context Assessment Scale (EACT).

The dependent variable was lumbar pain extracted from NMQ, self-reported and identified by a figure that specified the location for its identification. The independent variables were sex, age (continuous quantitative) and categorized by age group, marital status, number of children, position, work shift, years of work, time on duty, other employment relationship, Body Mass Index (BMI) satisfactory and unsatisfactory environmental conditions that include temperature, physical space, furniture, hygiene, sanitary facilities, resting place and food, work accidents, feeling of overload, bad mood, tiredness or fatigue at the end of the working day, factors that cause job insecurity, lack of job security, lack of autonomy, poor work environment, work performed, relationship with the boss, lack of training, work overload, inadequate facilities (bathrooms, changing rooms, etc.) and salaries. In order to determine BMI, the self-referred weight and height were classified as low / adequate <25 kg/m ^2^ , overweight ≥25 and <30 kg/m ^2^ and obese ≥30 and ≥40 kg/m ^2^ .

The NMQ identifies musculoskeletal pain or discomfort in the last twelve months as well as in the last seven days in the anatomical areas shown by a figure. The Brazilian version ^16^ presented good reliability and the Kappa coefficient values were at least 0.75 for each questionnaire item. It is an important instrument for identifying the most prevalent pain, specific anatomical area and disability in performing activities. The variable low back pain was dichotomized based on the pooled responses of the NMQ and meant no (not and rarely) and yes (often and always). This instrument was chosen because it is used worldwide in epidemiological studies with several populations, including nursing, in addition to being simple and easy to apply. It is a form of standardization between the researches and favors the comparison of the results.

The EACT was developed in 2003 and validated later between 2004 and 2006. This scale surveys people’s perceptions of their work context highlighting the critical points ^17^ . The basis of the scale is the concept of the Production Context of Goods and Services that unites multiple and diversified variables in a totality and makes an organizational diagnosis ^18^ . It examines three interdependent dimensions of work organization, working conditions and socio-professional relations. The working conditions dimension is expressed by the quality of the physical environment, work place, equipment and material made available for the work execution, being composed by ten items. The organizational dimension of work expresses the division of tasks, norms, controls and work rhythms, taking 11 items. The socio-professional relations dimension expresses the modes of work management, communication and professional interaction, being composed of ten items. The responses use a Likert scale ranging from 1 (never) to 5 (always) ^17 - 18^ .

The database was formed in the Microsoft Excel program. The variables underwent descriptive analysis. The categorical ones were submitted to the analysis of absolute and relative frequency and, finally, the continuous ones, by the means and standard deviation (SD). The analysis was performed in the Statistical Package for Social Sciences (SPSS), version 23.0 (IBM Corp., Armonk, United States). The chi-square test was used with dichotomization of the categorical variables and the odds ratio (OR) and its 95% confidence interval (95% CI). This association was also made in the binary logistic regression in the crude and adjusted analysis. The p value used was p<0.05. The confounding variables were selected from the literature, because they were related to the outcome. The input method of the variables in the regression model was the ENTER method (“forced entry”). The significance of the model was performed by Omnibus tests (p<0.05) and quality by the Hosmer and Lemeshow test (p>0.05), the residue analysis values were within the range of ± 2.5. The distance from Cook and DFBeta to constant were also checked to verify possibly influential cases (considering values greater than 1). The confounding factors considered were adjusted for age, sex, marital status, shift, post, years of work, Body Mass Index, hour on duty, other link.

In the EACT data analysis, the sum of the values assigned to each item is calculated to obtain the mean. The interpretation of the results was based on the classification of disease risk established by the authors of the instrument as satisfactory (1-2,3), critical (2,4-3,7) and severe (3,8-5,0) ^17 - 18^ . The satisfactory degree translates a positive result and well-being in the work, being this an aspect that must be maintained and consolidated in the organizational environment. The critical grade demonstrates a median outcome, indicating a limiting situation, evidencing a malaise at work and risk of illness. The serious degree shows a negative result and indicates malaise at work. Thus, there is a strong risk of illness and requires immediate measures in the causes of the diseases, with a view to eliminating and/or attenuating them ^17^ .

The research project was approved by the Research Ethics Committee involving Human Subjects under Opinion number 2,081,192 / 2017 and CAAE. 64164717.1.0000.0121.

## Results

Regarding the sociodemographic characteristics, the female sex predominated, being 83.4% of the sample; 66.4% are married or live with partners and 68.1% have children. The mean BMI was 26.34 kg/m ^2^ (SD 4.54) with a minimum of 17.97 and a maximum of 46.71, corresponding to the category of overweight, according to the World Health Organization, for both sexes. Just over half (52.5%) is above normal BMI. The overweight category corresponded to 34.6% of the sample, despite having a predominance of 41.9% with normal weight. The mean age was 41.12 years (SD 8.94), with a minimum of 22 years and a maximum of 64 years (fashion 38 years). When categorized by age group, a percentage of 30 years or more (10%), 31 years or more (34.5%), 41 to 50 years (32.2%) and 51 or more (23.3%).

Regarding labor variables, the auxiliary / nursing technician category corresponded to 79.4% and nurses totaled 20.6%. In relation to the years of work, the highest frequency (36.5%) was one to four years. The majority of the participants (78.4%) had 12 hour shifts, being (46.2%) during the day and (31.6%) at night, (76.4%) doing hour duty and (72.1%) have no other employment relationship. The working day at the institution is 30 hours / week, however, if you consider those who do hour on duty (overtime), the journey increases to an average of 50 hours a week.

Prevalence of pain or discomfort in the lumbar region, in the last 12 months (51.4%) and in the last week (45.4%), reached the highest indices in relation to the other regions of the body and was followed by the shoulder (46.1%), cervical region with 40.9%, and hip (39.7%) in the last 12 months; cervical region (40.3%), shoulders (34.7%) and hip (33.3%) in the last week. Approximately 85% of workers reported having at least one musculoskeletal symptom. In general, the prevalence of pain in other regions was also well reported. Regarding the limitation of the Daily Life Activities (ADL) due to the musculoskeletal symptom in the lumbar region, in the last 12 months, 81.9% had no limitation; (18.1%), followed by hip (14.1%) and cervical region (14.1%).

In the analysis of the environmental conditions of the workplace, classified as yes (satisfactory) or not (unsatisfactory), most considered as unsatisfactory almost all items: temperature (66.2%), inappropriate space (71%), furniture (78.5%), sanitary facilities (78.2%), rest (85%), except hygiene conditions that a little more than half of the respondents (52.5%) considered satisfactory. About 48% of the workers have already had an accident at work, the most common being a sharpshooter. Most respondents did not recognize workplace hazards or did not respond. Among the participants’ suggestions for improving working conditions was, in particular, increasing the number of human resources.

When responding to the questioning of how the worker feels at the end of the day, a variable also classified as yes or no, the feeling of being overwhelmed (p = 0.001), moody (p = 0.000) and fatigued (p = 0.002) presented a statistically significant association with lower back pain ( Table 1 ). Of those who say they are grumpy, fatigued and overwhelmed 83.3%, 70.4%, 67.9%, have lower back pain, respectively. By the analysis adjusted for confounding factors (age, sex, marital status, shift, post, years of work, BMI, hour on duty, other bond), feeling annoyed, fatigued and overwhelmed at the end of the day increased 6.38 (95% CI 2.00-20.33), 3.45 (95% CI 1.64-7.25) and 3.13 (95% CI 1.62-6.05) respectively, the chances of having backache. Regarding environmental conditions, the unsatisfactory furniture conditions had a significant association with low back pain and increased the chances of having low back pain by 2.20 (95% CI 1.13-4.27). Among the factors that cause job dissatisfaction, lack of recognition (p = 0.036), poor working environment (p = 0.023) and overload (p = 0.000) were associated with lower back pain, but in Binary Logistic Regression it was only associated with overload that increased 2.69 (95% CI 1.41-5.13) times the chances of presenting low back pain ( Table 1 ).

**Table 1:** Factors associated with nonspecific low back pain in the last twelve months in nursing workers of a public hospital in Florianópolis, SC, Brazil, 2017

Variables	Lumbar pain		Gross OR*(CI) ^†^	Ajusted OR ^‡^ (CI) ^†^	p value ^§^

	No f (%)	Yes f (%)			
Overload sensation					0,001
No	109 (54.5)	91 (45.5)	1	1	
Yes	25 (32.1)	53 (67.9)	2.53 (1.46-4.40)	3.13 (1.62-6.05) ^||^	
Feeling of bad mood					<0.001
No	129 (52.0)	119 (48.0)	1	1	
Yes	5 (16.7)	25 (83.3)	5.42(2.01-14.61)	6.38 (2.00-20.33) ^||^	
Feeling fatigued					0.002
No	118 (52.7)	106 (47.3)	1	1	
Yes	16 (29.6)	38 (70.4)	2.64 (1.39-5.01)	3.45 (1.64-7.25) ^||^	
Furniture					0.006
Unsatisfactory	94 (44.1)	119 (55.9)	2.24 (1.24-4.04)	2.20 (1.13-4.27) ^||^	
Satisfactory	39 (63.9)	22 (36.1)	1	1	
Lack of recognition					0.036
No	57 (57.0)	43 (43.0)	1	1	
Yes	74 (43.8)	95 (56.2)	1.70 (1.03-2.80)	1.78 (0.99-3.20)	
Bad environment					0.023
No	103 (52.8)	92 (47.2)	1	1	
Yes	28 (37.3)	47 (62.7)	1.87 (1.08-3.24)	1.46 (0.78-2.73)	
Overload					<0.001
No	55 (67.9)	26 (32.1)	1	1	
Yes	77 (40.5)	113 (59.5)	3.10 (1.79-5.37)	2.69 (1.41-5.13) ^||^	

*Gross OR = Odds Ratio analysis by univariate logistic regression; ^†^ CI = 95% Confidence Interval; ^‡^ Adjusted OR = Odds Ratio analysis with confounding variables, age, sex, marital status, shift, post, years of work, Body Mass Index, time on duty, other link with entry into the model by the enter method multivariate logistic regression; the significance of the model by the Omnibus tests (p0.05) and the quality by the Hosmer and Lemeshow test (p>0.05); the residue analysis values were within the range of ±2.5; ^§^ P value = level of significance p<0.05; ^ǁ^ Results of the adjusted analysis = there are significant differences between the studied variables

Regarding the overall mean of all EACT dimensions, the value was 3.2, classified as critical, with unsatisfactory working conditions and moderate risks for illness. The averages of each dimension and risk classification are shown in Table 2 .

**Table 2:** Descriptive statistics referring to the three dimensions of EACT* and risk classification for illness in nursing workers of a public hospital in Florianópolis, SC, Brazil, 2017

Dimensions of EACT*	Average	Standard deviation	Situation ^†^
Organization of work	3.49	±1.19	Critical
Work conditions	3.56	±1.22	Critical
Socio-professional relations	2.55	±1.22	Critical

*EACT = Work Context Assessment Scale; ^†^ Situation = Risk classification for illness

In the work organization dimension, the items “the tasks are repetitive” and the “work rhythm is excessive” and in the “working conditions” dimension the item “existing furniture in the workplace is inadequate” obtained a severe classification, which indicates risk of becoming ill and requires immediate action. The only items considered satisfactory belong to the dimension “socio-professional relations” and were “there are difficulties in the communication between manager and subordinates”, “the information that I need to perform my tasks are difficult to access” and “lack of support of the bosses for my professional development “.

The “working conditions” dimension (p = 0.007) and “work organization” (p = 0.004) showed a statistically significant association with low back pain ( Table 3 ). In the “organization of work”, the situation considered as severe increased by 9.06 the chances of presenting low back pain; in “working conditions,” the situation also classified as severe increased by 3.46 times the chance of having low back pain.

**Table 3:** Association between the dimensions of EACT* and nonspecific low back pain in the last 12 months in nursing workers of a public hospital in Florianópolis, SC, Brazil, 2017

Variable	Lumbar pain		Gross OR ^†^ (CI) ^‡^	Ajusted OR ^§^ (CI) ^‡^	p-value ^ǁ^

	No f (%)	Yes f (%)			
Work Organization					0.004
Satisfactory	8 (80.0)	2 (20.0)	1	1	
Critical	73 (55.7)	58 (44.3)	3.17 (0.65-15.54)	3.74 (0.64-21.71)	
Severe	33 (37.5)	55 (62.5)	6.66 (1.33-33.30)	9.06 (1.48-55.22) ^¶^	
Work conditions					0.007
Satisfactory	13 (72.2)	5 (27.8)	1	1	
Critical	60 (54.5)	50 (45.5)	2.16 (0.72-6.49)	1.63 (0.48-5.52)	
Severe	47 (39.2)	73 (60.8)	4.03 (1.33-12.06)	3.46 (1.01-11.85) ^¶^	
Socio-professional relations					0.071
Satisfactory	54 (58.1)	39 (41.9)	1	1	
Critical	50 (42.4)	68 (57.6)	1.88 (1.08-3.26)	2.07 (1.05–4.07) ^¶^	
Severe	8 (44.4)	10 (55.6)	1.73 (0.62-4.78)	1.90 (0.62-5.82)	

*EACT = Work Context Assessment Scale; ^†^ Gross OR = Odds Ratio analysis by univariate logistic regression; ‡CI = 95% confidence interval; ^§^ Adjusted OR = Odds Ratio analysis with confounding variables, age, sex, marital status, shift, post, years of work, Body Mass Index, time on duty, other link with entry into the model by the enter method multivariate logistic regression; significance of the model by Omnibus tests (p<0.05) and quality by the Hosmer and Lemeshow test (p>0.05); the residue analysis values were within the range of ±2.5; ^ǁ^ P value = Significance level p<0.05; ^¶^ Results of the adjusted analysis = There are significant differences between the studied variables

## Discussion

It was attempted to associate low back pain with the work process in a hospital unit with a sample of 301 nursing workers. The approach was innovative because the combination of these instruments to contextualize occupational low back pain was not found in the literature. It is important to emphasize the importance of research based on the experience of workers, considering that these generate knowledge, information and investigate the social organization in the workplace and help in the planning and execution of actions aimed at the prevention of health problems ^3 , 18^ .

The high prevalence of reports of pain symptoms and musculoskeletal discomfort in the lumbar region in this study demonstrates the impact of this problem on the nursing team and expresses a negative influence on the health of these workers. It is, therefore, an important concern in nursing practice and the risk factors of lumbar algias should be identified and resolved with priority ^19^ . Other national and international surveys also showed a high prevalence of low back pain in nursing with rates reaching 85.9% ^10 , 13 , 20 - 25^ .

Labor burdens such as overload, fatigue and moodiness (physiological and psychic workloads) were related to pain. High physical or mechanical demands stress and fatigue the muscles and can initiate a process of low back pain by prolonged positions and repetitive movements. The relationship between fatigue and pain seems to encompass metabolic and structural lesions that influence pain physiology-related channels such as the basal glands, thalamus, limbic system, and cortical center ^26^ . Psychosocial factors such as bad mood at the end of the journey and fatigue can influence chronicity, frequency, perception and pain threshold ^27^ .

Fatigue seems to play an important role in the etiology of psychophysical overloads. It can be conceptualized as a development of a feeling of physical and mental tiredness that modifies alertness and alertness, affects the ability to work and perform tasks. It can progress to chronic fatigue, increasing susceptibility to occupational diseases. In this sense, the poor working conditions increase the risks ^28^ . The study that evaluated the prevalence of musculoskeletal discomfort, work capacity and fatigue in nursing professionals, in addition to the high prevalence of musculoskeletal discomfort, showed greater fatigue and the need for rest, conditions that directly influenced the ability to work ^28^ . Another study found a statistically significant association between musculoskeletal disorders and chronic occupational fatigue among nurses and the need for preventive measures ^29^ .

In the hospital unit studied, the large portion of the servers is a nursing professional. Its service is organized with centralization of decisions and division of tasks between nurses and nursing technical personnel. Some tasks are individualized, others are performed with the help of another worker. They involve repetition of activities and movements and generate the same wear for a while. They are subject to prolongation of the journey by the relay in shifts, double bond or overtime and exposed to a wide variety of workloads. The multi-causality of low back pain is justified. In this sense, the work process may be a cause for injuries such as low back pain and other musculoskeletal disorders (MSD) related to contact with patients, exercise of procedures and type of task developed ^30^ .

The dimensions of EACT “work organization” and “working conditions” had a statistically significant association with lower back pain and a critical classification with a moderate potential for workers’ illness, which demands the taking of measures in the medium and short term. Similar results were found in a study with primary care workers ^18^ . Another study that analyzed the risks of illness of the nursing professional related to the work context in a psychiatric hospital also found a critical classification in the “work organization” dimension, but serious in the “working conditions” ^17^ . When precarious and subject to various workloads, authors describe working conditions and their organization as strong factors of illness ^4 , 9 , 14 , 30^ and nursing is exposed to precarious conditions in a chronic way ^30 - 31^ . One study found that work activities favor lumbar MSD and shows the importance of an occupational control with organizational, technical and individual measures ^32^ .

In the dimension “work organization”, the items “excessive work rate” and “tasks are repetitive”, which are physiological and psychic workloads, presented a serious risk classification. Studies that analyzed the risks of illness of the nursing worker related to the work context in a psychiatric hospital found a severe classification in the item repetitiveness of tasks ^17^ and was the item with the worst evaluation with nursing workers of the hemodialysis service ^33^ . Another study revealed a high prevalence of lumbar pain related to some tasks, including repetitive movements, and also found an association between lumbar complaints and absenteeism ^32^ . Repeated tasks cause tiredness and fatigue and feelings of boredom and anger that are enhanced by pressure for productivity and time to perform activities ^17 , 33^ .

The pace of work can be intensified by the prolongation of the workload represented by the rotation of shifts, overtime or association with another employment relationship. Although, in the present research, the majority do not have another job, the increase of the journey is associated to the fact of doing overtime and shifts. Another study showed that the longer daily workload and the large number of patients attended increase the occupational health risks. The low back pain group worked longer hours and spent more time walking or standing, which increased the pain risk by 35% for each additional hour worked ^19^ . There is no health security limit that can be established regarding the length of a work day. The fight for special conditions through regulation in a maximum of 30 hours a week leads to a safer practice of care, strengthening of the profession, as well as reducing exposure to risk factors ^34^ .

The extension of the journey is the extraction of surplus value, it is surplus expenditure of the labor force ^1 , 35^ . The capital steals the worker hours for healthy development and maintenance of the body, mealtime and rest; it breaks the moral and physical limits and there can be an exhaustion of this work force and the shortening of its life time. To further prolong the working day, shifts are organized, day and night ^1 , 35^ . This exploitation leads to physical and mental exhaustion, a result of the specific characteristics of the surplus value extraction strategy, the relationship between the social and the biopsychic ^3^ . Among the physical consequences may be occupational low back pain. The extension of the journey also covers the search for economy by the managers and increase of the productivity. Workers, in turn, earn extra pay in wages, but lose in the wear and tear experienced by increased exposure to labor burdens ^34^ .

As for the “working conditions” dimension, the inadequate furniture (mechanical work load), item with critical classification, potentiates the ergonomic risks; also highlighted in another study in a psychiatric hospital ^17^ and primary care ^18^ . In the studied institution, one could observe inadequacy in relation to materials, physical space and equipment. The nursing team performs its activities in limited spaces, the cabinets are improperly positioned, either very high or low, requiring inappropriate postures, beds with stuck cranks, and stretchers without height adjustments. Many pieces of furniture are deficient in health care facilities. Consequently, they favor the process of sickness of workers ^31^ . In addition, repetitive tasks, also with critical risk rating, associated with inadequate furniture and lack of space lead to ergonomic risk ^31^ . The lack of materials and equipment, maintenance of furniture and inadequate infrastructure enhance the physical burden ^14^ .

The “socio-professional relations” dimension, despite a critical risk assessment, did not present a statistically significant association with low back pain. No factor obtained severe evaluation. The factors “there are difficulties in communication between managers and subordinates”, “the information that I need to perform my tasks are difficult to access”, “lack of support from managers for my professional development”, which were considered satisfactory. This is a beneficial aspect in the scenario studied because the ease of communication and support exert a positive influence on the work environment. On the contrary, lack of support and integration in interpersonal relations leads to competitiveness, conflicts, emotional distractions and work overload that can bring unpleasant physical repercussions ^17^ . In studies, psychiatric hospital, hemodialysis service and emergency mobile service, socio-professional relations were evaluated as satisfactory ^17 , 33 , 36^ .

The results of the present study show how the work organization and its conditions may reflect the appearance and maintenance of low back pain in nursing workers in the studied health institution. Research shows that this is a reality of other jobs in which the conditions and work organization can influence the genesis of the diseases ^4 , 31^ .

The global process studied involves many causes and determinants that influence the work-health relationship. We sought to establish the biopsychic nexus that is the concrete expression of human corporality within the historical process at a given moment. In some of these moments, it can be identified as disease, defining a pathological profile. To establish it in a collective, it is necessary to analyze the work process and its conditions until its expression in the body of the workers ^6^ . The internal materiality was expressed by the occurrence of nonspecific low back pain that was manifested by the interaction between the loads.

The wear and tear that can lead to aggravations, even though they acquire individual corporeality, must be considered alongside reproductive processes in capitalist societies and can be measured by signs and symptoms that are not specific to the pathological profile and other indicators ^3 , 6^ . The interrelationship of organizational factors and working conditions in this study are not isolated risks, but they act dynamically with each other, they add up and become more potent because they were generated under certain conditions ^4 , 14^ . There are many workloads in this scenario, emphasizing the physiological, psychic and mechanical that have been related to low back pain.

Many workers are not aware of this exposure as demonstrated by the results of this research. Lack of control of occupational hazards can lead to accidents and illness. There is also a lack of protection and promotion of health by employers’ institutions ^30^ . This lack of risk perception is a comfortable condition desired by the responsible institutions. On the other hand, even if workers are aware of the risks, they must maintain constant and permanent struggles for better structured working conditions as a collective practice ^30^ .

Wear involves the loss of the capacity to develop initiatives and take control, it is the systematic denial of the creativity of the collective worker. Therefore, the process of attrition can interrupt the struggle of opposition to the loss of biopsychic capacities and the development of their potentialities. On the other hand, proper management of their activities will make them less fragile, with the form of workers gaining control over their own lives ^3^ . Care should be taken in the exposure to various workloads that can lead to illness to identify them early and to develop prevention strategies, since it is also possible to reverse losses and capacities ^6 , 9^ .

As a limitation of this study, it is verified that the studied variables involve subjectivity and self-reports. Your results should be analyzed with caution. Further analysis of the influence of the organization and working conditions on the occurrence of low back pain, how workers analyze critical outcomes, and what the suggestions for strategies to overcome problems through qualitative research would be relevant. Another limitation refers to the transversal nature of the study in which it is not possible to establish a relationship between cause and effect. Also, the NMQ questionnaire has some problems in its format, such as the type of question that includes, besides pain, symptoms of discomfort, numbness and the lack of an evaluation of the degree of severity of the symptoms, using only its occurrence as the risk load.

However, the study showed a statistically significant association between the conditions and organization of work with the outcome of low back pain. The same raised fragile aspects of these dimensions that can contribute to the aggravation in the context of work studied and with possibility of being happening in other work environments of nursing performance submitted to similar workloads.

The occurrence of lower back pain as a health problem of these workers contributed to show the need for monitoring and surveillance. Their results demonstrate the importance of investing in the scenario of nursing practice and improving their conditions.

## Conclusion

In this research, conditions classified as critical and serious in the work context, expressed by the nursing team, were identified, related to the conditions and work organization. It has been shown that factors such as duration of the journey, rhythms, deadlines, productivity, physical environment, equipment and instruments can have an important repercussion, being able to cause physical and mental exhaustion, such as low back pain and other injuries.

The “working conditions” and “work organization” dimensions were associated with low back pain with critical risk classification and cause deficits that potentiate the loads, especially the physiological, psychic and mechanical.

It was possible to analyze the hospital nursing work process and its articulations to the factors related to low back pain and the consequent health implications of the worker. For this, it was verified that the reflections must go beyond the physical causes and give visibility to the organization and working conditions that can generate illnesses and suffering to the worker.

It is hoped to contribute to a greater knowledge in the health of the worker regarding low back pain and to broaden the discussion about the work process in nursing and strengthening of the profession. Similar new research promises relevant contributions to health and nursing.
